# Prognostic significance of hemoglobin A1c level in patients hospitalized with coronary artery disease. A systematic review and meta-analysis

**DOI:** 10.1186/1475-2840-10-98

**Published:** 2011-11-10

**Authors:** Yao Liu, Yan-min Yang, Jun Zhu, Hui-qiong Tan, Yan Liang, Jian-dong Li

**Affiliations:** 1 Institution of all authors: Emergency Department, Cardiovascular Institute and Fuwai Hospital; Chinese Academy of Medical Sciences & Peking Union Medical College, (167 Beilishilu Road), Beijing, (100037), China

**Keywords:** hemoglobin A1c, mortality, coronary artery disease, acute coronary syndrome

## Abstract

**Background:**

The prognostic value of hemoglobin A1c (HbA1c) in coronary artery disease (CAD) remains controversial. Herein, we conducted a systematic review to quantify the association between elevated HbA1c levels and all-cause mortality among patients hospitalized with CAD.

**Methods:**

A systematic search of electronic databases (PubMed, EMBASE, OVID, Web of Science, The Cochrane Library) for studies published from 1970 to May 2011 was performed. Cohort, case-control studies, and randomized controlled trials that examined the effect of HbA1c on all-cause mortality were included.

**Results:**

Twenty studies met final inclusion criteria (total n = 13, 224). From the pooled analyses, elevated HbA1c level was significantly associated with increased short-term (OR 2.32, 95% CI, 1.61 to 3.35) and long-term (OR 1.54, 95% CI, 1.23 to 1.94) mortality risk. Subgroup analyses suggested elevated HbA1c level predicted higher mortality risk in patients without diabetes (OR 1.84, 95% CI, 1.51 to 2.24). In contrast, in patients with diabetes, elevated HbA1c level was not associated with increased risk of mortality (OR 0.95, 95% CI, 0.70 to 1.28). In a risk-adjusted sensitivity analyses, elevated HbA1c was also associated with a significantly high risk of adjusted mortality in patients without diabetes (adjusted OR 1.49, 95% CI, 1.24 to 1.79), but had a borderline effect in patients with diabetes (adjusted OR 1.05, 95% CI, 1.00 to 1.11).

**Conclusions:**

Our findings demonstrate that elevated HbA1c level is an independent risk factor for mortality in CAD patients without diabetes, but not in patients with established diabetes. Prospective studies should further investigate whether glycemic control might improve outcomes in CAD patients without previously diagnosed diabetes.

## Background

In recent years, much attention has been paid to the glycometabolism in patients with coronary artery disease (CAD). Numerous prior studies have shown that elevated admission or fasting glucose increases the risk of death and in-hospital complications in patients with acute coronary syndrome (ACS) and patients undergoing coronary revascularization [1-5].

Hemoglobin A_1c _(HbA_1c_) level is an indicator of average blood glucose concentrations over the preceding 2-3 months, which is a convenient and well-known biomarker in clinical practice. Epidemiological evidence now suggests that HbA1c level is an independent risk factor for cardiovascular events in primary and secondary populations [6-9]. Recently, an International Expert Committee Report (IECR) recommended using the HbA1c assay as the preferred method for diabetes diagnosis and suggested the diagnosis if the HbA1c level is ≥6.5% [10]. However, the prognostic value of HbA1c level in patients with coronary atherosclerotic disease has not been well characterized, and these studies that examined this relationship have reported conflicting results [11-23].

To comprehensively analyze these data, we performed a systematic review to examine whether an association exists between elevated HbA1c and all-cause mortality in patients hospitalized with CAD.

## Methods

The methods for this meta-analysis are in accordance with "Meta-Analysis of Observational Studies in Epidemiology: A Proposal for Reporting." [24]

### Search strategy

A systematic search of publications listed in the electronic databases (Medline via PubMed, EMBASE, OVID, Web of Science, The Cochrane Library) from 1970 to May 2011 were conducted using the following key words in combination as both MeSH terms and text words: ("coronary artery disease" or "acute coronary syndrome" or "acute myocardial infarction" or "percutaneous coronary intervention" or "coronary artery bypass grafting") and ("glycated hemoglobin" or "hemoglobin A1c" or "HbA1c"). Language restrictions were not applied, but our search was limited to human studies. The list of articles was reviewed independently by two authors. In addition, a manual review of references from primary or review articles was performed to identify any additional relevant studies

### Study selection

Cohort, case-control studies, and randomized controlled trials were included if they investigated the influence of HbA1c on all-cause mortality in patients admitted with CAD. The IECR recommended that HbA_1c _level > 6.5% would be the cut-off value for diagnosis of diabetes [10]. In patients with diabetes, the American Diabetes Association (ADA) recommended HbA_1c _< 7% is associated with a lower risk of diabetes-associated complications [25]. We anticipated that not all studies would use HbA1c value 6.5% or 7% as the cut-off point. Therefore, in order to avoid eliminating studies with important information, we considered HbA1c cut-off within the range of 5% - 8% to be acceptable.

After obtaining full reports of candidate studies, the same reviewers independently assessed eligibility. Differences in data between the two reviewers were resolved by reviewing corresponding articles, and the final set was agreed on by consensus. If the publications did not contain the full information necessary for meta-analysis, we obtained the missing information directly from the authors (see Acknowledgments).

### Quality assessment and data abstraction

Each study was evaluated for quality according to the guidelines provided by the United States Preventive Task Force [26] and published recommendations [27]. The following characteristics were assessed: (1) description of patient sample characteristics; (2) clear inclusion and exclusion criteria; (3) potential selection bias; (4) a priori definition of study outcomes; (5) completeness of follow-up; (6) adjustment of possible confounders in multivariate analysis; (7) explanation of sample selection; and (8) timing of determination (whether HbA1c measured at baseline). Studies were graded as poor quality if they met ≤4 criteria, fair if they met 5 to 6 criteria, and good if they met ≥7 criteria.

For each study, the following data were extracted: first author's last name, the publication year, study design, the sample size, study population, baseline characteristics, length of follow-up, timing of HbA1c measurement, cut-off value of HbA1c, variables adjusted for in the multivariate statistical analysis, mortality data among patients with and without elevated HbA1c level, and adjusted mortality hazard ratios (HRs) with their 95% CIs if possible.

### Statistical analyses

We used REVMAN software (version 5.0; Cochrane Collaboration, Oxford, United Kingdom) and Stata software (version 11.0; Stata Corporation, College Station, TX) to pool data for all outcomes. Firstly, unadjusted short and long term all-cause mortality data were extracted and pooled to calculate odds ratio (OR) and 95% CI using a random effects model for dichotomous outcomes. Relative risks were also calculated separately for diabetic and non-diabetic patients when possible. Secondly, we performed baseline risk-adjusted analyses to determine if our main results were robust when quantitative pooling was limited to those studies in which we could calculate pooled adjusted all-cause mortality ORs. Heterogeneity was assessed visually using Cochran's χ^2 ^statistics and the I^2 ^statistics. A random-effect model of DerSimonian and Laird was applied to calculate overall differences. Publication bias was estimated using a funnel plot of study results against study precision. We tested symmetry of the funnel plot using the Egger's test. P value was considered statistically significant at < 0.05.

## Results

### Study selection

Our initial search yielded 1883 potential literature citations. Of these, 1844 were excluded after scanning titles and abstracts, leaving 39 citations for full text assessment. Of these, 20 studies with a total of 13, 224 patients met the inclusion criteria and were used for this meta-analysis [13-23,28-36]. The remaining studies were excluded largely because they did not include mortality as an outcome or did not provide mortality data according to HbA1c level; other subjects were included besides CAD patients; or reported HbA1c was in an unusable format (mean and SDs for patients with and without mortality). Additional data were requested from the authors of three studies [11,12,19] but obtained for only one study [19].

### Characteristics of the trials

Baseline characteristics of the 20 studies included are shown in Additional file 1 and six studies were retrospective [19,22,28,32,33,36]. We found no randomized controlled trial that examined the effect of HbA1c on mortality in patients admitted with CAD. Demographic features of study populations (age, gender) were similar across the studies, and the average age of the patients ranged from 56 to 75 years. The study population included 5258 patients (40%) admitted with AMI or ACS, 4948 (37.4%) patients who underwent CAGB and 2990 patients (22.6%) underwent PCI. Five of the 20 studies investigated short-term (less than 3 months) all-cause mortality [28-30,34,36], 13 studies reported long-term (1 year to 8 years) mortality [13-20,22,23,32,33,35] and 2 studies with both short and long-term follow-up results [21,31]. All studies were of high methodological quality (good or fair) (Additional file 1).

Table 1 shows baseline characteristics and treatment of the patients in original articles according to HbA1c level. As expected, patients with elevated HbA1c level had a higher prevalence of comorbidities including hypertension, heart failure, previous MI and renal insufficiency. Use of angiotensin-converting enzyme (ACE) inhibitors, β-blockers and lipid lowering drugs was higher in patients with elevated HbA1c compared to those with normal HbA1c level.

**Table 1:** Baseline characteristics of the study population according to HbA1c

Baseline Characteristics	Patients With Elevated HbA1c	Patients With Normal HbA1c
Age (y), studies = 12, n = 10734	64	65
Male (%), studies = 12, n = 10734	64	72
History of Hypertension (%), studies = 10, n = 10242	74	59
History of Heart failure (%), studies = 4, n = 5276	22	14
Previous MI (%), studies = 3, n = 4219	48	24
Hyperlipidemia (%), studies = 3, n = 4149	36	35
Smoker (%), studies = 7, n = 3770	40	41
Body mass index (kg/m2), studies = 6, n = 2990	27	29
Renal insufficiency (%), studies = 5, n = 5806	10	7
β-Blocker (%), studies = 4, n = 3794	77	77
Angiotensin-converting enzyme (ACE) inhibitors (%), studies = 3, n = 1448	53	39
Lipid lowering drugs (%), studies = 4, n = 3794	67	41

Six studies [13,16,18,21,22,28] exclusively examined non-diabetic patients, and the other six studies studied diabetes alone [14,15,32,33,35,36], the percent of diabetic patients was not reported in two studies [19,30] and the rest incorporated patients with and without diabetes [17,20,23,29,31,34]. Diabetes status was assigned on the basis of a history of diabetes or treatment with hypoglycemic agents in 10 studies [14,15,17,20,23,29,31,32,34,35]; in three of these studies, an elevated HbA1c [35] or plasma glucose [14,17,35] was also used to define newly diagnosed diabetes for patients without diabetes history. The definition of diabetes was not specified in two studies [33,36].

### HbA1c and risk of mortality

**Figure 1:**
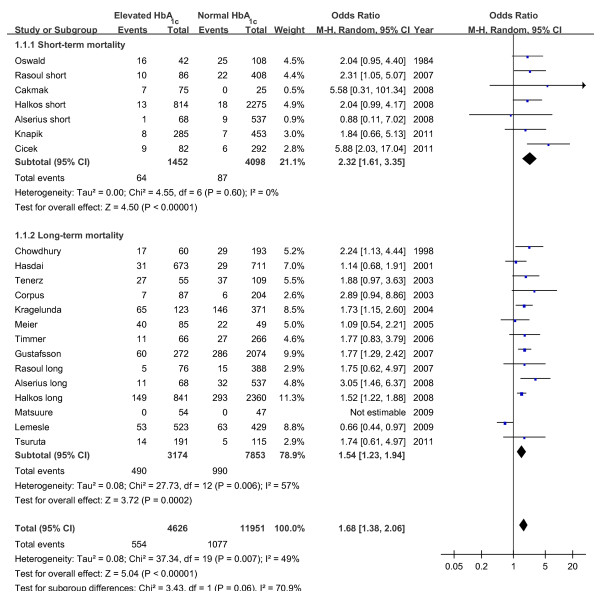
**Unadjusted risk of short and long term mortality based on elevated or normal HbA1c level**.

Nineteen studies reported all-cause mortality data according to HbA1c levels [13,15-23,28-36] (Figure 1). Analyses of the crude mortality revealed that 14.1% of patients with elevated HbA1c level died compared with 11.7% of those with normal HbA1c. This translated into an unadjusted mortality risk of OR 1.68 (95% CI, 1.38 to 2.06) in patients with elevated HbA1c. There was moderate statistical heterogeneity among the studies (I^2 ^= 49%, p < 0.001). We found no evidence of publication bias based on funnel plot or using the Egger's test (P = 0.089). Subgroup analyses showed in the short-term follow up cohort, the elevated HbA1c level was associated with a more than two-fold increased risk of mortality (OR 2.32, 95% CI, 1.61 to 3.35), with no evidence for overall heterogeneity (I^2 ^= 0%, p = 0.48). Whereas patients with higher HbA1c level had a 54% increased risk of long-term mortality (OR 1.54, 95% CI, 1.23 to 1.94). And there was no significant difference between the short-term and long-term results (p = 0.06 for subgroup difference).

**Figure 2:**
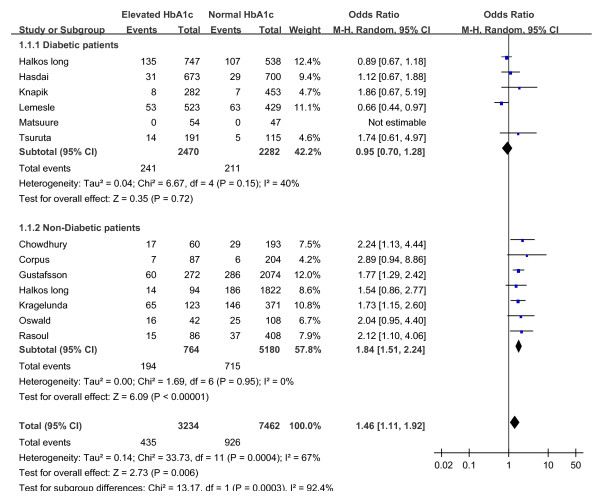
**Unadjusted risk of mortality for patients with or without diabetes based on elevated or normal HbA1c level**.

In addition, we performed a subgroup analyses to compare the association between HbA1c and mortality in patients with and without diabetes. These analyses were restricted to the 12 studies in which separate mortality data for diabetic and non- diabetic patients were reported [13,15,16,18,21-23,28,32,33,35,36] (Figure 2). In patients without diabetes, elevated HbA1c was associated with a 84% increased risk of mortality (OR 1.84, 95% CI, 1.51 to 2.24). While in patients with diabetes, elevated HbA1c level was not associated with a significantly higher risk of mortality (OR 0.95, 95% CI, 0.70 to 1.28).

### Risk adjusted analyses

**Figure 3:**
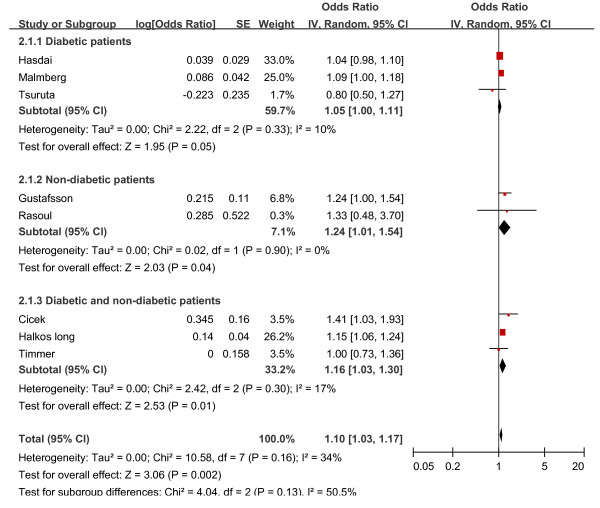
**Adjusted mortality risk ratio for elevated HbA1c level**.

Eight studies used a Cox proportional hazards regression model to adjust for other prognostic factors to determine independent association of HbA1c with mortality [14,15,20-23,34,35] (Figure 3). All studies adjusted for age and gender. Other covariates adjusted for included diabetes [14,20,23], hypertension [14,15,22,23], hyperlipidemia [14,20,22,33], heart failure [14,15,22,23], smoker [14,15,22,23,35], arrhythmia [22,23], previous CAD [14,15,20,22], multivessel disease [15,20,21], renal function [22,23,35], peripheral vascular disease [23], and study treatment [22]. In patients without diabetes, the pooled analyses suggested elevated HbA1c was independently associated with mortality after multivariate adjusted (adjusted OR 1.24, 95% CI, 1.01 to 1.54). In the three studies combined data for diabetic and nondiabetic patients, HbA1c level was also a predictor of mortality independent of other prognostic factors (adjusted OR 1.16, 95% CI, 1.03 to 1.30). But elevated HbA1c was not significantly associated with adjusted risk of death in diabetic patients (adjusted OR 1.05, 95% CI, 1.00 to 1.11). The overall pooled adjusted relative risk for elevated HbA1c was OR, 1.10 (95% CI, 1.03 to 1.17) for all the eight studies.

## Discussion

The results of our meta-analysis suggest that elevated HbA1c levels predict increased risk of short and long term mortality in patients hospitalized with CAD. Subgroup analyses showed that the impact of HbA1c on mortality seemed to be different between patients with and without diabetes. Elevated HbA1c was associated with a higher risk of mortality in patients without recognized diabetes even after adjusting for other known risk factors, but had a neutral effect on mortality in patients with diabetes.

Diabetes mellitus and acute glycemic control (admission, fasting or preoperative glucose level) are independent prognostic factors for survival following ACS or AMI [1-5,37,38]. Although acute hyperglycemia for most patients may be a manifestation of antecedent disturbed glucose metabolism [1,2], it is also partly caused by the transient stress with release of catecholamines and cortisol [39,40]. The adverse outcomes associated with acute hyperglycemia may be somewhat attributed to the stress response to a severe disease state. HbA1c level is a stable indicator of unstressed long-term glucose control and is more useful to predict the abnormal glucose tolerance in CAD patients compared with admission glucose [41]. However, recent studies that evaluated the prognostic value of HbA1c in patients hospitalized with advanced atherosclerosis have reported discrepant results. Several studies showed that although crude mortality data was higher in patients with elevated HbA1c following adjustment for many cardiovascular risk factors, HbA1c values failed to predict mortality independently [11,15,16,20,30,36]. Others suggested that HbA1c level was a potent predictor of in-hospital and long-term death [13,14,21,23,29,31,34]. After systematically reviewing previous published data and directly comparing the effect of HbA1 on outcomes in patients with and without recognized diabetes, we found that HbA1c levels had different prognostic effects based on patient's diabetes status.

There are several reasons for the discrepant findings between patients with and without diabetes. First, patients with elevated HbA1c but without known diabetes likely have diabetes that was neither diagnosed nor treated, and other relevant cardiovascular risk factors such as hypertension and dyslipidemia that were also untreated before hospitalization; while those with diabetes are more likely to be treated with insulin and control the established risk factors [2,4]. This therapeutic difference may account in part for the disparity in outcomes. Second, the same cutoff value that defined elevated HbA1c may have been relatively too low to distinguish those with chronic hyperglycemia in diabetic patients compared with in non-diabetic patients. Third, recent studies suggested progress in the reperfusion treatment of patients with AMI improved the outcome of diabetic patients [42]. Effective reperfusion especially PCI might attenuate the adverse effect of elevated HbA1c on outcomes of diabetic patients [15]. Fourth, HbA1c may lose its predictive power in diabetic patients with heart failure as it has been recognized for other cardiovascular risk factors like BMI and total cholesterol [43]. A recent study reported the association between mortality and HbA1C among 5815 diabetic patients with heart failure appeared U-shaped, HbA1c ≤ 7.1 and > 7.8% all associated with higher risk of death [44]. Another cohort study in diabetic patients with advanced heart failure observed an inverse relationship between HbA1c and long-term mortality [45]. Heart failure was present in ~20% of the diabetic patients included in our analysis. Future studies should investigate the impact of HbA1c in different high-risk diabetic populations with CAD. Fifth, the UK Prospective Diabetes Study (UKPDS) showed that although intensive glycemic treatment resulted in a non-significant improvement in macrovascular disease during the 5-year trial period [46], the 10-year follow-up demonstrated a significant risk reduction for myocardial infarction and death emerged over time [47]. The follow-up periods for diabetic patients in our study were all less than five years. Therefore, if the follow-up periods were longer, more differences in the mortality between diabetic patients with and without elevated HbA1c might have emerged.

Elevated HbA1c level is likely the result of long term insulin resistance; metabolic disturbances associated with insulin resistance including hyperglycemia, dyslipidemia, hypercoagulability and inflammation might the major pathologic mechanism for the adverse impact of elevated HbA1c in the setting of CAD [48]. In risk-adjusted analysis, after adjusted for other prognostic factors such as old age, hypertension, prior myocardial infarction, and hyperlipidaemia, HbA1c remained independently associated with increased mortality risk (except for patients with diabetes), but it was observed that the associations were attenuated. This suggested that the adverse effect of HbA1c on mortality is somewhat attributable to a correlation with other cardiovascular risk factors. The presence of an elevated HbA1c level, through its association with the metabolic syndrome, may potentially predate the development of other risk factors, and hence these risk factors may represent a common biological proatherogenic pathway [49,50].

Prior clinical trials of intensive glucose control have shown little benefit, and possibly some harm, of lowering the HbA1c level in patients with diabetes to prevent cardiovascular outcomes [46,47,51,52]. The results of two recent meta-analyses suggested that intensive glucose control reduced cardiovascular events with no effect on mortality [53,54], and subgroup analyses showed participants with a history of cardiovascular disease did not appear to benefit from intensive therapy [54]. The majority of current trials for glycemic control were designed primarily to patients with established diabetes. Our observation support the need for large randomized trials designed to confirm or refute preliminary suggestions that intensive glycemic control may improve outcomes in CAD patients without diabetes and elevated HbA1c level.

Several limitations of this meta-analysis should be noted. First, the pooled studies differed in inclusion and exclusion criteria, cutoffs for elevated HbA1c, definition of diabetes, duration of follow-up and concomitant treatment. These may be the major source of heterogeneity. We used a random effects model in an effort to incorporate heterogeneity between trials in our analysis, but recognize that this does not eliminate the fact that heterogeneities were present. Second, given the lack of data in some studies, less than half the studies were combined for adjusted analysis, which meant that the results of risk-adjusted analysis were less conclusive. And the covariates adjusted in each study were different. Given the limitations, the results should be interpreted cautiously. Third, although we found no significant effect of elevated HbA1c on all-cause mortality in patients with diabetes, recent studies suggested that HbA1c was associated with an increased risk of major adverse cardiac events following stent implantation [55] and was a predictor of ischemic events in diabetic patients [56]. The prognostic effect of HbA1c on other cardiovascular events in diabetic patients with CAD needs further evaluation. Finally, given that a proportion of studies included are retrospective, a possibility of residual confounding by unmeasured factors cannot be eliminated. This provided associative, not causal, evidence and mandates caution when interpreting these results.

## Conclusions

In conclusion, we found that HbA1c level is an independent predictor of total mortality in CAD patients without but not in patients with established diabetes. Glycemic control, diabetes screening, and risk factor modification may represent opportunities to improve care in this group of patients.

## Competing interests

The authors declare that they have no competing interests.

## Authors' contributions

YL and YMY contributed to study concept and design, search of the literatures, data extraction, data analyses, and the drafting and review of the final manuscript. JZ contributed to the conception and design of the analysis, interpreted the analyzed data, critically reviewed the manuscript, and helped to draft the manuscript. HQT, YL, and JDL participated in design of the analysis, data interpretation, and review of the manuscript. All authors read and approved the final manuscript.

## Supplementary Material

Additional file 1 **Characteristics of selected studies**. Additional file 1 shows baseline characteristics of the 20 studies included. Click here for file

## References

[B1] Kosiborod M Rathore SS Inzucchi SE Masoudi FA Wang Y Havranek EP Krumholz HM Admission glucose and mortality in elderly patients hospitalized with acute myocardial infarction: implications for patients with and without recognized diabetes Circulation 2005 111 3078 3086 10.1161/CIRCULATIONAHA.104.517839 15939812

[B2] Deedwania P Kosiborod M Barrett E Ceriello A Isley W Mazzone T Raskin P American Heart Association Diabetes Committee of the Council on Nutrition, Physical Activity, and Metabolis Hyperglycemia and acute coronary syndrome: a scientific statement from the American Heart Association Diabetes Committee of the Council on Nutrition, Physical Activity, and Metabolism Circulation 2008 117 1610 9 10.1161/CIRCULATIONAHA.107.188629 18299505

[B3] Suleiman M Hammerman H Boulos M Kapeliovich MR Suleiman A Agmon Y Markiewicz W Aronson D Fasting glucose is an important independent risk factor for 30-day mortality in patients with acute myocardial infarction: a prospective study Circulation 2005 111 754 760 10.1161/01.CIR.0000155235.48601.2A 15699267

[B4] Straumann E Kurz DJ Muntwyler J Stettler I Furrer M Naegeli B Frielingsdorf J Schuiki E Mury R Bertel O Spinas GA Admission glucose concentrations independently predict early and late mortality in patients with acute myocardial infarction treated by primary or rescue percutaneous coronary intervention Am Heart J 2005 150 1000 6 10.1016/j.ahj.2005.01.033 16290985

[B5] Imran SA Ransom TP Buth KJ Clayton D Al-Shehri B Ur E Ali IS Impact of admission serum glucose level on in-hospital outcomes following coronary artery bypass grafting surgery Can J Cardiol 2010 26 151 4 10.1016/S0828-282X(10)70357-3 20352135 PMC2851469

[B6] Stratton IM Adler AI Neil HA Matthews DR Manley SE Cull CA Hadden D Turner RC Holman RR Association of glycaemia with macrovascular and microvascular complications of type 2 diabetes (UKPDS 35): prospective observational study BMJ 2000 321 405 12 10.1136/bmj.321.7258.405 10938048 PMC27454

[B7] Selvin E Steffes MW Zhu H Matsushita K Wagenknecht L Pankow J Coresh J Brancati FL Glycated hemoglobin, diabetes, and cardiovascular risk in nondiabetic adults N Engl J Med 2010 362 800 11 10.1056/NEJMoa0908359 20200384 PMC2872990

[B8] Eeg-Olofsson K Cederholm J Nilsson PM Zethelius B Svensson AM Gudbjörnsdóttir S Eliasson B New aspects of HbA1c as a risk factor for cardiovascular diseases in type 2 diabetes: an observational study from the Swedish National Diabetes Register (NDR) J Intern Med 2010 268 471 82 10.1111/j.1365-2796.2010.02265.x 20804517

[B9] Nishimura R Nakagami T Sone H Ohashi Y Tajima N Relationship between hemoglobin A1c and cardiovascular disease in mild-to-moderate hypercholesterolemic Japanese individuals: subanalysis of a large-scale randomized controlled trial Cardiovasc Diabetol 2011 10 58 10.1186/1475-2840-10-58 21714932 PMC3150244

[B10] The International Expert Committee International Expert Committee Report on the role of the A1C assay in the diagnosis of diabetes Diabetes Care 200 32 1327 1334 10.2337/dc09-9033 PMC2699715 19502545

[B11] Hadjadj S Coisne D Mauco G Ragot S Duengler F Sosner P Torremocha F Herpin D Marechaud R Prognostic value of admission plasma glucose and HbA in acute myocardial infarction Diabet Med 2004 21 305 310 10.1111/j.1464-5491.2004.01112.x 15049930

[B12] Cao JJ Hudson M Jankowski M Whitehouse F Weaver WD Relation of chronic and acute glycemic control on mortality in acute myocardial infarction with diabetes mellitus Am J Cardiol 2005 96 183 186 10.1016/j.amjcard.2005.03.040 16018838

[B13] Chowdhury TA Lasker SS Elevated glycated haemoglobin in non-diabetic patients is associated with an increased mortality in myocardial infarction Postgrad Med J 1998 74 480 1 10.1136/pgmj.74.874.480 9926122 PMC2360891

[B14] Malmberg K Norhammar A Wedel H Rydén L Glycometabolic state at admission: important risk marker of mortality in conventionally treated patients with diabetes mellitus and acute myocardial infarction: long-term results from the Diabetes and Insulin-Glucose Infusion in Acute Myocardial Infarction (DIGAMI) study Circulation 1999 99 2626 32 10338454 10.1161/01.cir.99.20.2626

[B15] Hasdai D Rizza RA Grill DE Scott CG Garratt KN Holmes DR Jr Glycemic control and outcome of diabetic patients after successful percutaneous coronary revascularization Am Heart J 2001 141 117 23 10.1067/mhj.2001.111957 11136496

[B16] Corpus RA O'Neill WW Dixon SR Timmis GC Devlin WH Relation of hemoglobin A1c to rate of major adverse cardiac events in nondiabetic patients undergoing percutaneous coronary revascularization Am J Cardiol 2003 92 1282 6 10.1016/j.amjcard.2003.08.008 14636904

[B17] Tenerz A Nilsson G Forberg R Ohrvik J Malmberg K Berne C Leppert J Basal glucometabolic status has an impact on long-term prognosis following an acute myocardial infarction in non-diabetic patients J Intern Med 2003 254 494 503 10.1046/j.1365-2796.2003.01221.x 14535972

[B18] Kragelund C Snorgaard O Køber L Bengtsson B Ottesen M Højberg S Olesen C Kjaergaard JJ Carlsen J Torp-Petersen C TRACE Study Group Hyperinsulinaemia is associated with increased long-term mortality following acute myocardial infarction in non-diabetic patients Eur Heart J 2004 25 1891 7 10.1016/j.ehj.2004.07.033 15522467

[B19] Meier JJ Deifuss S Klamann A Launhardt V Schmiegel WH Nauck MA Plasma glucose at hospital admission and previous metabolic control determine myocardial infarct size and survival in patients with and without type 2 diabetes: the Langendreer Myocardial Infarction and Blood Glucose in Diabetic Patients Assessment (LAMBDA) Diabetes Care 2005 28 2551 3 10.2337/diacare.28.10.2551 16186299

[B20] Timmer JR Ottervanger JP Bilo HJ Dambrink JH Miedema K Hoorntje JC Zijlstra F Prognostic value of admission glucose and glycosylated haemoglobin levels in acute coronary syndromes QJM 2006 99 237 43 10.1093/qjmed/hcl028 16504985

[B21] Rasoul S Ottervanger JP Bilo HJ Timmer JR van 't Hof AW Dambrink JH Dikkeschei LD Hoorntje JC de Boer MJ Zijlstra F Glucose dysregulation in nondiabetic patients with ST-elevation myocardial infarction: acute and chronic glucose dysregulation in STEMI Neth J Med 2007 65 95 100 17387235

[B22] Gustafsson I Kistorp CN James MK Faber JO Dickstein K Hildebrandt PR OPTIMAAL Study Group Unrecognized glycometabolic disturbance as measured by hemoglobin A1c is associated with a poor outcome after acute myocardial infarction Am Heart J 2007 154 470 6 10.1016/j.ahj.2007.04.057 17719292

[B23] Halkos ME Lattouf OM Puskas JD Kilgo P Cooper WA Morris CD Guyton RA Thourani VH Elevated preoperative hemoglobin A1c level is associated with reduced long-term survival after coronary artery bypass surgery Ann Thorac Surg 2008 86 1431 7 10.1016/j.athoracsur.2008.06.078 19049726

[B24] Stroup DF Berlin JA Morton SC Olkin I Williamson GD Rennie D Moher D Becker BJ Sipe TA Thacker SB Meta-analysis of observational studies in epidemiology: a proposal for reporting: Meta-Analysis of Observational Studies in Epidemiology (MOOSE) group JAMA 2000 283 2008 2012 10.1001/jama.283.15.2008 10789670

[B25] American Diabetes Association. Standards of medical care in diabetes Diabetes Care 2005 28 Suppl 1 S4 S36 15618112

[B26] Harris RP Helfand M Woolf SH Lohr KN Mulrow CD Teutsch SM Atkins D Methods Work Group, Third US Preventive Services Task Force Current methods of the US Preventive Services Task Force: a review of the process Am J Prev Med 2001 20 21 35 11306229 10.1016/s0749-3797(01)00261-6

[B27] Altman DG Systematic reviews of evaluations of prognostic variables BMJ 2001 323 224 8 10.1136/bmj.323.7306.224 11473921 PMC1120839

[B28] Oswald GA Corcoran S Yudkin JS Prevalence and risks of hyperglycaemia and undiagnosed diabetes in patients with acute myocardial infarction Lancet 1984 1 1264 7 6144976 10.1016/s0140-6736(84)92447-4

[B29] Halkos ME Puskas JD Lattouf OM Kilgo P Kerendi F Song HK Guyton RA Thourani VH Elevated preoperative hemoglobin A1c level is predictive of adverse events after coronary artery bypass surgery J Thorac Cardiovasc Surg 2008 136 631 40 10.1016/j.jtcvs.2008.02.091 18805264

[B30] Cakmak M Cakmak N Cetemen S Tanriverdi H Enc Y Teskin O Kilic ID The value of admission glycosylated hemoglobin level in patients with acute myocardial infarction Can J Cardiol 2008 24 375 8 10.1016/S0828-282X(08)70600-7 18464942 PMC2643139

[B31] Alserius T Anderson RE Hammar N Nordqvist T Ivert T Elevated glycosylated haemoglobin (HbA1c) is a risk marker in coronary artery bypass surgery Scand Cardiovasc J 2008 42 392 8 10.1080/14017430801942393 18609043

[B32] Matsuura K Imamaki M Ishida A Shimura H Niitsuma Y Miyazaki M Off-pump coronary artery bypass grafting for poorly controlled diabetic patients Ann Thorac Cardiovasc Surg 2009 15 18 22 19262445

[B33] Lemesle G Bonello L de Labriolle A Maluenda G Syed AI Collins SD Ben-Dor I Torguson R Kaneshige K Xue Z Suddath WO Satler LF Kent KM Lindsay J Pichard AD Waksman R Prognostic value of hemoglobin A1C levels in patients with diabetes mellitus undergoing percutaneous coronary intervention with stent implantation Am J Cardiol 2009 104 41 5 10.1016/j.amjcard.2009.02.060 19576319

[B34] Cicek G Uyarel H Ergelen M Ayhan E Abanonu GB Eren M Gibson CM Hemoglobin A1c as a prognostic marker in patients undergoing primary angioplasty for acute myocardial infarction Coron Artery Dis 2011 22 131 7 10.1097/MCA.0b013e328342c760 21394027

[B35] Tsuruta R Miyauchi K Yamamoto T Dohi S Tambara K Dohi T Inaba H Kuwaki K Daida H Amano A Effect of preoperative hemoglobin A1c levels on long-term outcomes for diabetic patients after off-pump coronary artery bypass grafting J Cardiol 2011 57 181 6 10.1016/j.jjcc.2010.11.003 21185154

[B36] Knapik P Cieśla D Filipiak K Knapik M Zembala M Prevalence and clinical significance of elevated preoperative glycosylated hemoglobin in diabetic patients scheduled for coronary artery surgery Eur J Cardiothorac Surg 2011 39 484 9 10.1016/j.ejcts.2010.07.037 21087870

[B37] Donahoe SM Stewart GC McCabe CH Mohanavelu S Murphy SA Cannon CP Antman EM Diabetes and mortality following acute coronary syndromes JAMA 2007 298 765 75 10.1001/jama.298.7.765 17699010

[B38] Kümler T Gislason GH Køber L Torp-Pedersen C Diabetes is an independent predictor of survival 17 years after myocardial infarction: follow-up of the TRACE registry Cardiovasc Diabetol 2010 9 22 10.1186/1475-2840-9-22 20525192 PMC2893120

[B39] Husband DJ Alberti KG Julian DG 'Stress'hyperglycaemia during acute myocardial infarction: an indicator of pre-existing diabetes? Lancet 1983 ii 179 181 10.1016/s0140-6736(83)90169-1 6135025

[B40] Monnier L Mas E Ginet C Michel F Villon L Cristol JP Colette C Activation of oxidative stress by acute glucose fluctuations compared with sustained chronic hyperglycemia in patients with type 2 diabetes JAMA 2006 295 1681 1687 10.1001/jama.295.14.1681 16609090

[B41] Ishihara M Inoue I Kawagoe T Shimatani Y Kurisu S Hata T Nakama Y Kijima Y Kagawa E Is admission hyperglycaemia in non-diabetic patients with acute myocardial infarction a surrogate for previously undiagnosed abnormal glucose tolerance? Eur Heart J 2006 27 2413 9 10.1093/eurheartj/ehl271 17000629

[B42] Hadjadj S Coisne D Mauco G Ragot S Duengler F Sosner P Torremocha F Herpin D Marechaud R Diabetes mellitus and outcome after primary coronary angioplasty for acute myocardial infarction: lessons from the GUSTO-IIb angioplasty substudy J Am Coll Cardiol 2000 35 1502 1512 10.1016/S0735-1097(00)00591-X 10807453

[B43] Gustafsson F Kragelund CB Torp-Pedersen C Effect of obesity and being overweight on long-term mortality in congestive heart failure: influence of left ventricular systolic function Eur Heart J 2005 26 58 64 15615800 10.1093/eurheartj/ehi022

[B44] Aguilar D Bozkurt B Ramasubbu K Deswal A Relationship of hemoglobin A1C and mortality in heart failure patients with diabetes J Am Coll Cardiol 2009 54 422 8 10.1016/j.jacc.2009.04.049 19628117 PMC2753214

[B45] Eshaghian S Horwich TB Fonarow GC An unexpected inverse relationship between HbA1c levels and mortality in patients with diabetes and advanced systolic heart failure Am Heart J 2006 151 91.e1 91.e6 10.1016/j.ahj.2005.10.008 16368297

[B46] UK Prospective Diabetes Study (UKPDS) Group Intensive blood-glucose control with sulphonylureas or insulin compared with conventional treatment and risk of complications in patientswith type 2 diabetes (UKPDS 33) Lancet 1998 352 837 853 9742976

[B47] Holman RR Paul SK Bethel MA Neil HA Matthews DR Long-term follow-up after tight control of blood pressure in type 2 diabetes N Engl J Med 2008 359 1565 76 10.1056/NEJMoa0806359 18784091

[B48] Bansilal S Farkouh ME Fuster V Role of insulin resistance and hyperglycemia in the development of atherosclerosis Am J Cardiol 2007 99 6B 14B 17307054 10.1016/j.amjcard.2006.11.002

[B49] DeFronzo RA Ferrannini E Insulin resistance: a multifaceted syndrome responsible for NIDDM, obesity, hypertension, dyslipidemia, and atherosclerotic cardiovascular disease Diabetes Care 1991 14 173 194 10.2337/diacare.14.3.173 2044434

[B50] Grundy SM Obesity, metabolic syndrome, and coronary atherosclerosis Circulation 2002 105 2696 2698 10.1161/01.CIR.0000020650.86137.84 12057978

[B51] Duckworth W Abraira C Moritz T Reda D Emanuele N Reaven PD Zieve FJ Marks J Davis SN Hayward R Warren SR Goldman S McCarren M Vitek ME Henderson WG Huang GD VADT Investigators Glucose control and vascular complications in veterans with type 2 diabetes N Engl J Med 2009 360 129 139 10.1056/NEJMoa0808431 19092145

[B52] Action to Control Cardiovascular Risk in Diabetes Study Group Gerstein HC Miller ME Byington RP Goff DC Jr Bigger JT Buse JB Cushman WC Genuth S Ismail-Beigi F Grimm RH Jr Probstfield JL Simons-Morton DG Friedewald WT Effects of intensive glucose lowering in type 2 diabetes N Engl J Med 2008 358 2545 59 18539917 10.1056/NEJMoa0802743 PMC4551392

[B53] Ray KK Seshasai SR Wijesuriya S Sivakumaran R Nethercott S Preiss D Erqou S Sattar N Effect of intensive control of glucose on cardiovascular outcomes and death in patients with diabetes mellitus: a meta-analysis of randomised controlled trials Lancet 2009 373 1765 72 10.1016/S0140-6736(09)60697-8 19465231

[B54] Control Group Turnbull FM Abraira C Anderson RJ Byington RP Chalmers JP Duckworth WC Evans GW Gerstein HC Holman RR Moritz TE Neal BC Ninomiya T Patel AA Paul SK Travert F Woodward M Intensive glucose control and macrovascular outcomes in type 2 diabetes Diabetologia 2009 52 2288 98 10.1007/s00125-009-1470-0 19655124

[B55] Ueda H Mitsusada N Harimoto K Miyawaki M Yasuga Y Hiraoka H Glycosylated hemoglobin is a predictor of major adverse cardiac events after drug-eluting stent implantation in patients with diabetes mellitus Cardiology 2010 116 51 7 10.1159/000314331 20453503

[B56] Camafort M Alvarez-Rodríguez LR Muñoz-Torrero JF Sahuquillo JC López-Jiménez L Coll R Monreal M FRENA Investigators Glucose control and outcome in patients with stable diabetes and previous coronary, cerebrovascular or peripheral artery disease. Findings from the FRENA Registry Diabet Med 2011 28 73 80 10.1111/j.1464-5491.2010.03153.x 21166848

