# Supplementary material for: Prognostic significance of hemoglobin A1c level in patients hospitalized with coronary artery disease. A systematic review and meta-analysis
Source: Cardiovasc Diabetol. 2011 Nov 10;10:98. doi: 10.1186/1475-2840-10-98 (PMC3225330; doi:10.1186/1475-2840-10-98)
Supplement: Additional file 1 — Characteristics of selected studies. Additional file 1 shows baseline characteristics of the 20 studies included. [file 1475-2840-10-98-S1.DOC]

**Additional file 1. Characteristics of selected studies**

| Author | Year | Study Design | No. | Diabetes, % | Mean age，y | Male, % | Patients | Timing of  HbA1c Measured | Follow-Up | HbA1c cutoff, % |
| --- | --- | --- | --- | --- | --- | --- | --- | --- | --- | --- |
| **Short term Follow-up cohorts** | | | | | |  | | |  |  |
| Oswald et al, [28] | 1984 | Retrospective | 150 | 0 | 62 | NA | AMI | Admission. | 3 months | 7.5 |
| Rasoul et al, [21] | 2007 | Prospective | 504 | 0 | 63 | 62 | STEMI | Admission. | 30d | 6.0 |
| Cakmak et al, [30] | 2008 | Prospective | 100 | NA | 60 | 68 | AMI | Admission. | 4 weeks | 6.5 |
| Halkos et al, [29]  (J Thorac cardio Surg) | 2008 | Prospective | 3089 | 40.1 | 63 | 73 | Undergoing  CABG | Preoperative | In hospital | 7 |
| Alserius et al, [31] | 2008 | Prospective | 605 | 26.6 | 66 | 79 | Undergoing  CABG | Preoperative | <30d | 6 |
| Knapik et al, [36] | 2011 | Retrospective | 735 | 100 | 65 | 66 | Undergoing  CABG | Preoperative | In hospital | 7 |
| Cicek et al, [34] | 2011 | Prospective | 374 | 36.4 | 56 | 85 | Undergoing  PCI | Admission. | In hospital | 6.5 |
| **Long term Follow-up cohorts** | | | |  | |  | | | | |
| Chowdhury et al, [13] | 1998 | Prospective | 301 | 0 | 64 | 60 | AMI | Admission. | 1 years | 6.1 |
| Malmberg et al*, [14] | 1999 | Prospective | 620 | 100 | 68 | 63 | AMI | Admission. | 3.4 years | NA |
| Hasdai et al, [15] | 2001 | Prospective | 1373 | 100 | 65 | 60 | Undergoing  PCI | Mean 3 days  after PCI | 3.2-4.7 years | 8.0 |
| Corpus et al, [16] | 2003 | Prospective | 291 | 0 | 66 | 74 | Undergoing  PCI | Admission. | 1 year | 6 |
| Tenerz et al, [17] | 2003 | Prospective | 305 | 24 | 75, | 63 | AMI | Admission. | 5.5 years | 5.0 |
| Kragelunda et al, [18] | 2004 | Prospective | 494 | 0 | 67 | 69 | AMI | Admission. | 6-8 years | 5.2 |
| Meier et al, [19] | 2005 | Retrospective | 134 | NA | NA | NA | AMI | Preoperative | 3.5 years | 7 |
| Timmer et al, [20] | 2006 | Prospective | 332 | 15 | 63 | 67 | ACS | Admission. | 1.6 years | 6.2 |
| Rasoul et al, [21] | 2007 | Prospective | 504 | 0 | 63 | 62 | STEMI | Admission. | 1.6 years | 6.0 |
| Gustafsson et al, [22] | 2007 | Retrospective | 2346 | 0 | 67 | 72 | MI | Admission. | 2.5 years | 5.2 |
| Halkos et al, [23]  (Ann thorac Surg) | 2008 | Prospective | 3201 | 40.1 | 63 | 72 | Undergoing  CABG | Preoperative | 5 years | 7 |
| Alserius et al, [31] | 2008 | Prospective | 605 | 26.6 | 66 | 79 | Undergoing  CABG | Preoperative | <30d and  3.5 year | 6 |
| Matsuure et al, [32] | 2009 | Retrospective | 101 | 100 | 65 | 79 | Undergoing  CABG | Preoperative | 2.2 years | 6.5 |
| Lemesle et al, [33] | 2009 | Retrospective | 952 | 100 | 65 | 60 | Undergoing  PCI | Preoperative | 1 years | 7 |
| Tsuruta et al, [35] | 2011 | Prospective | 306 | 100 | 60 | 79 | Undergoing  CABG | Preoperative | 3.6 years | 6.5 |

AMI, acute myocardial infarction; PCI, percutaneous coronary intervention; ACS, acute coronary syndrome; STEMI, ST-segment elevation myocardial infarction; CABG, coronary artery bypass grafting; NA, not applicable.

* Only adjusted mortality odds ratio and 95% CI was reported in the study by Malmberg et al [12].
